# Optimization and validation of echo times of point-resolved spectroscopy for cystathionine detection in gliomas

**DOI:** 10.1186/s40644-024-00764-x

**Published:** 2024-09-02

**Authors:** Min Zhou, Zhuang Nie, Jie Zhao, Yao Xiao, Xiaohua Hong, Yuhui Wang, Chengjun Dong, Alexander P. Lin, Ziqiao Lei

**Affiliations:** 1 Department of Radiology, Union Hospital, Tongji Medical College, Huazhong University of Science and Technology, Wuhan, Hubei China; 2 Center for Clinical Spectroscopy, Brigham and Women’s Hospital, Harvard Medical School, Boston, MA USA; 3 Tumor Center, Union Hospital, Tongji Medical College, Huazhong University of Science and Technology, Wuhan, Hubei China

**Keywords:** Magnetic resonance spectroscopy, Point-resolved spectroscopy, 1p/19q codeletion, Glioma, Cystathionine

## Abstract

**Background:**

Cystathionine accumulates selectively in 1p/19q-codeleted gliomas, and can serve as a possible noninvasive biomarker. This study aims to optimize the echo time (TE) of point-resolved spectroscopy (PRESS) for cystathionine detection in gliomas, and evaluate the diagnostic accuracy of PRESS for 1p/19q-codeletion identification.

**Methods:**

The TE of PRESS was optimized with numerical and phantom analysis to better resolve cystathionine from the overlapping aspartate multiplets. The optimized and 97 ms TE PRESS were then applied to 84 prospectively enrolled patients suspected of glioma or glioma recurrence to examine the influence of aspartate on cystathionine quantification by fitting the spectra with and without aspartate. The diagnostic performance of PRESS for 1p/19q-codeleted gliomas were assessed.

**Results:**

The TE of PRESS was optimized as (TE1, TE2) = (17 ms, 28 ms). The spectral pattern of cystathionine and aspartate were consistent between calculation and phantom. The mean concentrations of cystathionine in vivo fitting without aspartate were significantly higher than those fitting with full basis-set for 97 ms TE PRESS (1.97 ± 2.01 mM vs. 1.55 ± 1.95 mM, *p* < 0.01), but not significantly different for 45 ms method (0.801 ± 1.217 mM and 0.796 ± 1.217 mM, *p* = 0.494). The cystathionine concentrations of 45 ms approach was better correlated with those of edited MRS than 97 ms counterparts (*r* = 0.68 vs. 0.49, both *p* < 0.01). The sensitivity and specificity for discriminating 1p/19q-codeleted gliomas were 66.7% and 73.7% for 45 ms method, and 44.4% and 52.5% for 97 ms method, respectively.

**Conclusion:**

The 45 ms TE PRESS yields more precise cystathionine estimates than the 97 ms method, and is anticipated to facilitate noninvasive diagnosis of 1p/19q-codeleted gliomas, and treatment response monitoring in those patients. Medium diagnostic performance of PRESS for 1p/19q-codeleted gliomas were observed, and warrants further investigations.

**Supplementary Information:**

The online version contains supplementary material available at 10.1186/s40644-024-00764-x.

## Background

Codeletion of chromosome arms 1p and 19q, which is specifically linked to the oligodendroglial histologic subtype, stands for 1 of the most important gene mutations in gliomas [1]. Gliomas harboring 1p/19q codeletion are associated with a more favorable prognosis and remarkable chemosensitivity [2]. Studies have shown that 1p/19q-codeleted gliomas have special cancer cell metabolism with possible therapeutic implications. A clear example is that 1p/19q codeletion induces heterozygous loss for serine- and glutathione-pathway genes located on chromosome 1p: phosphoglycerate dehydrogenase and cystathionine gamma-lyase, leading to accumulation of cystathionine, an immediate precursor of cysteine and thus glutathione, and abnormal levels of other metabolites [3]. Those changes point to a possible selective vulnerability of 1p/19q-codeleted gliomas to serine and glutathione depletion. Thus, precise measurement of cystathionine, especially in vivo, would be of great importance in accurate identification of 1p/19q-codeleted gliomas and monitoring treatment response in these patients, and highlighted the need for advanced imaging techniques.

Magnetic resonance spectroscopy offers a noninvasive technique to examine the concentration of metabolites in brain tumors. The cystathionine molecule contains 8 non-exchangeable protons from ^2^CH, ^3^CH_2_, ^4^CH_2_, ^6^CH_2_, and ^7^CH groups, giving rise to multiplets at approximately 4 locations at 3T due to the coupling connections of spins: 2.2, 2.7, 3.1, and 3.8 ppm [4]. The signal at 2.72 ppm, which arises from the H4 and H4′ spins, is expected to be a characteristic spectral pattern for cystathionine because of the close resonance of H4 and H4′, and less spectral overlap with adjacent resonances [4, 5]. With the significant advances in MRS technique, a few in-vivo MRS studies of cystathionine have been reported recently. Branzoli et al. reported the first detection of cystathionine in vivo by MRS in gliomas, using J difference editing of the 2.72 ppm resonance [3]. However, point-resolved spectroscopy (PRESS) sequence is the most commonly accessible technique for MRS on clinical scanners, offering quite straightforward methods for data analysis and enabling the quantification of full metabolic profile in tumors. Thus measurement of cystathionine by optimized PRESS holds significant potential for clinical utility. Branzoli et al. also tried in vivo detection of cystathionine with 97 ms echo time (TE) PRESS method which was optimized for the detection of 2-hydroxyglutarate, a specific oncometabolite for IDH-mutated gliomas [5–7]. Their results demonstrated that omission of cystathionine from spectra analysis caused significant differences in the quantification of several metabolites, with aspartate the most affected, showing a 2.69-fold higher concentration, and the concentrations of aspartate even significantly correlated with those of cystathionine when fitting with full basis set, with the correlation coefficient of -0.53 ± 0.09. This is mainly because the multiplets of aspartate, in proximity of 2.7 and 3.8 ppm, both extensively overlap with cystathionine signals, and may therefore lead to wrong evaluation of cystathionine levels in gliomas. Of note, both the cystathionine and aspartate resonances are scalar coupled, resulting in variations in the spectral pattern and signal strength as the TE of PRESS sequence changes. This feature allows for the optimization of TE to improve the differentiation between the overlapping resonance signals.

Considering the potential role of cystathionine detection by MRS as an important biomarker in clinical management of glioma patients, and the lack of prior studies on optimization of PRESS for cystathionine detection, in this study, we try to establish the best TE of PRESS sequence for detection of cystathionine with computer simulations, and then validate it in both phantom analysis and clinical patients with brain masses.

## Methods

### Optimization of TE of PRESS for cystathionine measurement

Computer simulations were performed using the product-operator-based transformation matrix algorithm, as previously described [8], to optimize the PRESS sequence TE for cystathionine detection at 3T. The simulations involved numerically calculating the time evolution of density operators for cystathionine and aspartate proton spins, incorporating the effects of 90° and 180° slice-selective radio-frequency and gradient pulses. A product-operator-based transformation matrix was constructed for each slice-selective radio-frequency pulse to represent the coherence evolution during NMR actions and used to compute the density operator at the onset of data acquisition across various echo times. Spectra for cystathionine and aspartate (ratio 1:1) were simulated across a range of PRESS subecho times (TE1, TE2) from 10 to 200 ms. A TE set that gave the largest discrepancy between cystathionine and aspartate multiplets and a pronounced cystathionine signal at 2.72 ppm was selected as the optimal TE set. The density matrix simulations were performed using the MATLAB-based toolkit FID-A with in-house modifications to align with the on-site scanner’s field strength (2.89 T at our site) and radio-frequency pulse time parameters [9]. Published chemical shifts and coupling constants for cystathionine and aspartate were used [3, 10]. T2 relaxation effects were ignored in the simulations to reduce computational complexity [8, 11]. 

Finally, the TE of PRESS was optimized as (TE1, TE2) = (17 ms, 28 ms) for cystathionine measurement, as given in Result section.

### Phantom experiments

We performed phantom experiments to establish that the simulated spectra of cystathionine and aspartate were calculated accurately. This was done using 2 spherical phantoms at pH = 7.0: phantom 1 composing of cystathionine (1mM) and glycine (5 mM), and phantom 2 consisting of aspartate (1mM) and glycine (5 mM). Glycine was added for chemical shift reference with the singlet at 3.55 ppm [8, 10, 11]. Phantom spectra were obtained using the same 97 ms and 45 ms TE PRESS sequences as in vivo acquisitions, with the parameters of TR = 2000 ms, TE = 97 and 45 ms, VOI = 20 × 20 × 20 mm^3^, 256 averages, spectral width = 2000 Hz, and 1024 points.

### Patient population

The protocol was approved by the Institutional Review Board of Union Hospital. We obtained written informed consent for each subject.

Patients with newly diagnosed brain mass suspicious of gliomas or previously treated glioma patients presenting with suspected recurrence were prospectively screened from January to September 2022. All patients met the following inclusion criteria: (1) age 18 years or older; (2) the visible tumor mass of at least 20 × 20 × 20 mm (axial, sagittal, coronal) on brain T2-FLAIR images to ensure sufficient tissue for MRS. Exclusion criteria included MRI contraindications or other serious medical conditions.

### Histological diagnosis and 1p/19q codeletion analysis

For patients who had surgical resection or diagnostic biopsy, the histological diagnosis and immunohistochemistry analyses were performed according to the standard clinical pathology procedures at our institution. 1p/19q codeletion status were determined by gene sequencing.

### MRI and MRS

All MRI and MRS exams were performed on one clinical 3T MRI scanner (Siemens TIM Skyra, VE11C) with a 32-channel head coil. Axial 3-dimensional T2-FLAIR (TR/TI/TE = 5000 ms/1650 ms/388 ms, FOV = 250 × 230.5 mm, matrix = 256 × 205) images were acquired prior to spectroscopy, and reconstructed in the sagittal and coronal planes with 2 mm slice resolution for accurate localization of the voxel.

The MRS protocol included a 97 ms TE single-voxel PRESS sequence, a 45 ms TE single-voxel PRESS sequence, and a single-voxel Mescher-Garwood point-resolved spectroscopy (MEGA-PRESS) sequence. All 3 spectra were acquired from a same voxel under the direction of a neuroradiologist (MZ) to include as much of the lesion as possible while avoiding cystic, hemorrhagic, or necrotic regions. Localized shimming was performed using the automatic three-dimensional B0 field mapping technique. When the full width at half maximum (FWHM) of the water signal was ≥ 14 Hz, manual shimming was performed to optimize the magnetic field homogeneity of the volume of interest to a line width of < 14 Hz FWHM. The variable power with optimized relaxation delays and outer volume suppression techniques was utilized for water suppression. Following each water-suppressed acquisition, an unsuppressed water scan was acquired for metabolite quantification and eddy current correction using the same gradient parameters except for average = 4.

For the 97 ms and 45 ms TE PRESS, the acquisition parameters included: TR = 2000 ms, TE = 97 and 45 ms, 128 averages, spectral width = 2000 Hz, 1024 points, and a total time = 4.26 min for each sequence. A default TE1, TE2 = (17 ms, 80 ms) was used for the 97 ms TE PRESS sequence in our scanner.

For the MEGA-PRESS sequence, spectra were acquired using previously described parameters (TR = 2000 ms, TE = 68 ms, 128 pairs of scans, total time = 8.50 min) [3]. The editing pulse was applied at 1.9 ppm for the edit-on condition and at 7.5 pm for the edit-off condition, in an interleaved fashion.

### MRS data processing

The PRESS raw data were processed for correction of frequency and phase using an in-house Python program [12]. LCModel software (Version 6.3-1R) was used for spectral fitting, using simulated spectra of metabolites as customized basis sets. The basis set for PRESS contained basis spectra of 25 metabolites, and MEGA-PRESS consisted of 8 metabolites (Additional File 1). The basis spectra were numerically calculated with MATLAB FID-A [9], incorporating the radio-frequency and gradient pulse waveforms identical to those used in the scanner.

For tumor data, the quantification was carried out by scaling the signal using the unsuppressed water reference, assuming a tumor bulk water concentration of 55.5 mM [5]. T2 relaxation effects were compensated using 150 ms for water, and previously reported T2 values for the metabolites [13–15]. The T1 relaxation times of water and the metabolites were similar and would therefore approximately cancel, so corrections were neglected. Cramer-Rao lower bounds (CRLB) were used for evaluating the precision of the metabolite estimates. PRESS spectra with an signal-to-noise ratio < 5 or FWHM of creatine peak > 0.143 ppm were excluded due to poor quality [6]. For MEGA-PRESS, spectra were visually inspected and excluded if noise obscured the spectra (other than N-acetylaspartate) such that it could not be reliably fitted.

For both 97 ms and 45 ms TE PRESS data, the LCModel fitting were repeated twice, with and without aspartate in the basis set, to compare the influence of aspartate on cystathionine quantification.

MRS acquisition and post-processing methods are summarized in Additional File 1 [16]. 

### Statistical analysis

A paired t-test was used to compare the cystathionine concentrations derived from the PRESS spectra fitted with the full basis set to those estimated without aspartate in the basis set. Also, the correlation between the 2 cystathionine measurements above, and the cystathionine concentrations obtained from the PRESS and MEGA-PRESS data were determined by Spearman correlation method. The Kruskal-Wallis test was applied to compare the cystathionine concentrations as well as the corresponding CRLBs of 1p/19q-codeleted gliomas, 1p/19q non-codeleted gliomas and other tumors. A significance level of 0.05 was utilized for statistical tests. All analyses were performed using SPSS (Version 21, IBM Corporation).

## Results

### Optimization of TE of PRESS for cystathionine detection

**Fig. 1:**
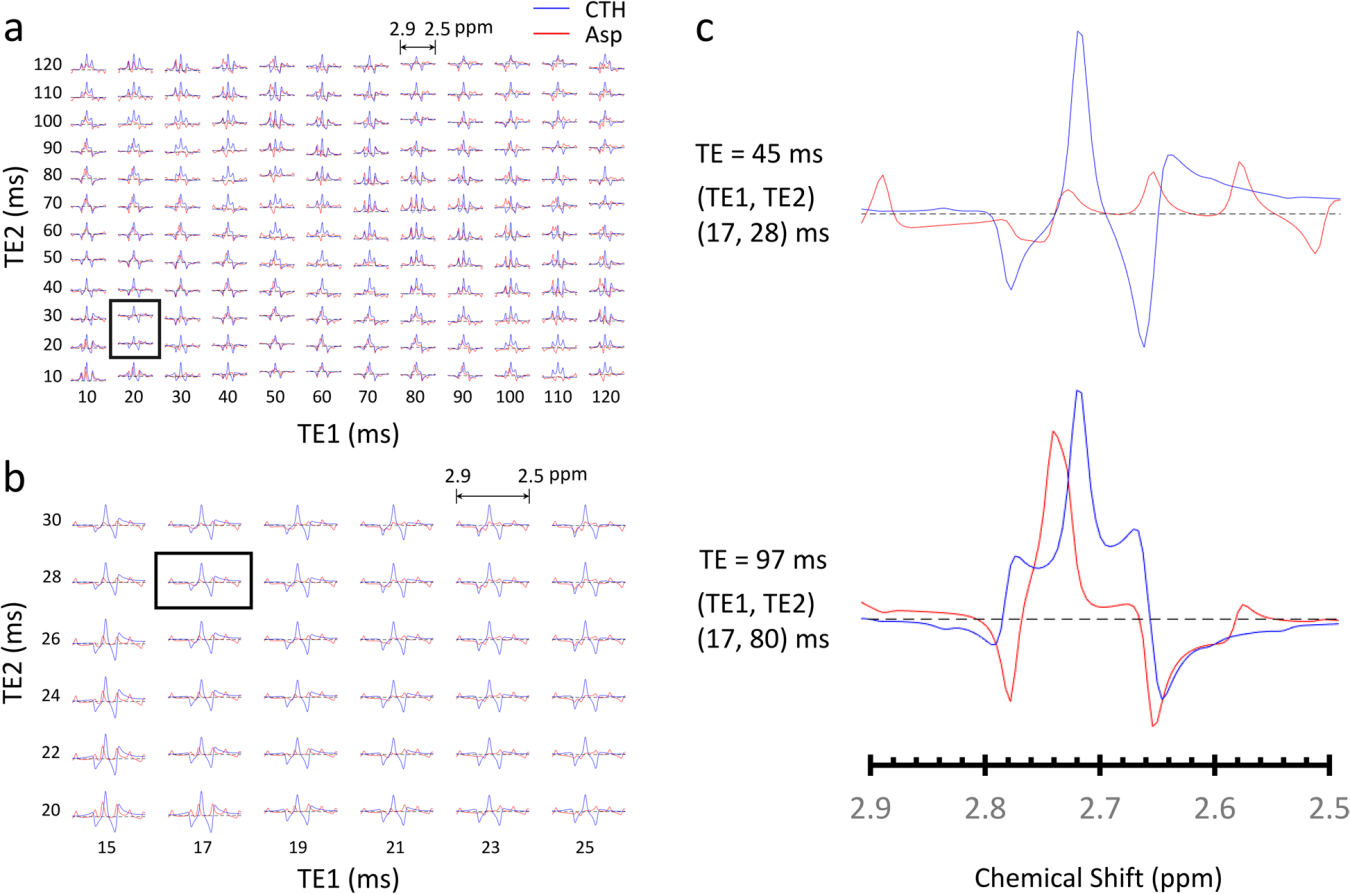
The numerical simulations of PRESS spectra of cystathionine and aspartate around 2.72 ppm: **a**, simulations with the TE1 and TE2 in a range of 10–120 ms with 10 ms increments. The cystathionine pattern is better visualized from aspartate peak with the subecho time sets around (TE1, TE2) = (20 ms, 20 ms) and (20 ms, 30 ms) (black box). **b**, further calculations with TE1 from 15 ms to 25 ms and TE2 between 20 ms and 30 ms in 2 ms increments. The TE set of PRESS is optimized as (TE1, TE2) = (17 ms, 28 ms) for cystathionine detection (black box). **c**, the cystathionine and aspartate spectra are shown with total TEs of 45 ms and 97 ms. CTH: cystathionine; Asp: aspartate

The numerical simulations of PRESS spectra of cystathionine and aspartate were performed in two steps. First, the simulations were conducted with the subecho times in a range of 10–120 ms with 10 ms increments (Fig. 1a). The simulations indicated that the subecho time sets around (TE1, TE2) = (20 ms, 20 ms) and (20 ms, 30 ms) gave rise to a larger discrepancy between cystathionine and aspartate peaks with a higher cystathionine peak at approximately 2.72 ppm. We then performed further simulations with TE1 from 15 ms to 25 ms and TE2 between 20 ms and 30 ms in 2 ms increments (Fig. 1b). The results showed that the aspartate multiples were relatively flat while the cystathionine resonances were pronounced around 2.72 ppm at a total TE of 45–49 ms, making it easier to resolve cystathionine from the background aspartate signal. The TE of PRESS sequence was optimized as (TE1, TE2) = (17 ms, 28 ms) for cystathionine detection in our study. The spectra of cystathionine and aspartate at TE = 45 ms vs. 97 ms are shown in Fig. 1c.

### Phantom experiments

**Fig. 2:**
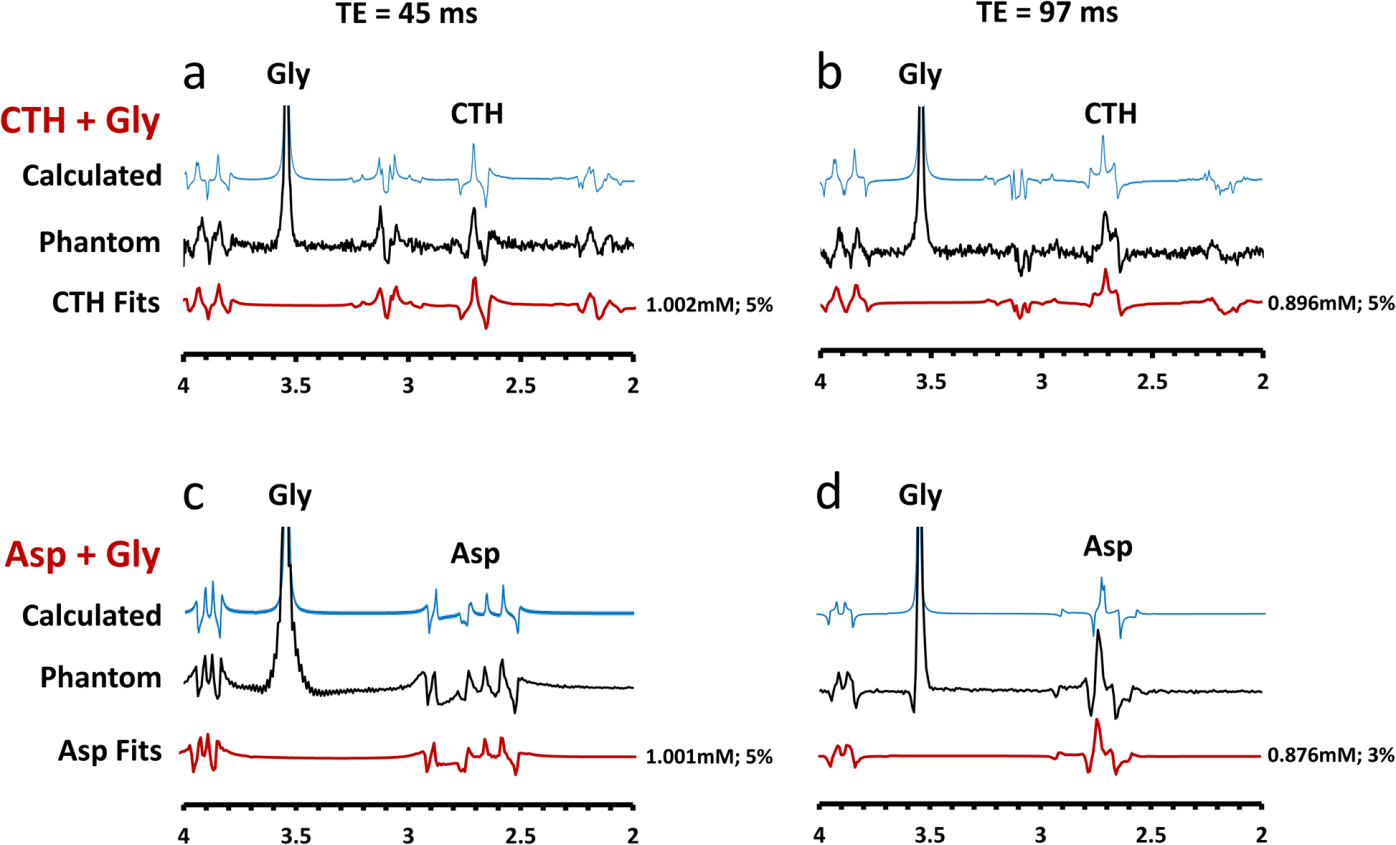
The simulated and phantom spectra of cystathionine + glycine (**a**, **b**) and aspartate + glycine (**c**, **d**) for PRESS with TEs of 45 ms and 97 ms. The spectra pattern of each phantom data are consistent with those of the calculated data. For the phantom scans, the absolute concentrations of cystathionine and aspartate by LCModel fitting are all in close agreement with the actual concentration of 1mM, and the small discrepancies are readily attributed to machine noise. CTH: cystathionine; Asp: aspartate; Gly: glycine

The simulated and phantom spectra of cystathionine (phantom 1) and aspartate (phantom 2) for PRESS with total TEs of 45 ms and 97 ms are shown in Fig. 2. The spectra pattern of each phantom data were consistent with those of the calculated data, confirming the accuracy of our computer simulations.

### Patient population

Eighty-five patients were enrolled. One patient was excluded due to poor spectra quality in both 97 ms and 45 ms TE PRESS scans, resulting in a total of 84 patients including in this study. Seventy-seven patients underwent surgery or biopsy, and 67 were diagnosed with gliomas (51 newly-diagnosed vs. 16 recurrent-glioma patients). Gene sequencing was carried out in 53 of the glioma patients, and 1p/19q codeletion were confirmed in 9 patients.

Eighty-four, 34 and 53 spectra were available for 97 ms TE PRESS, 45 ms TE PRESS and MEGA-PRESS, respectively. The numbers were different either because 45 ms TE PRESS and MEGA-PRESS were not routine practice and not performed in some patients due to busy clinical schedule, or because PRESS (*n* = 1) or MEGA-PRESS (*n* = 6) was excluded due to not meeting the quality control criteria.

**Fig. 3:**
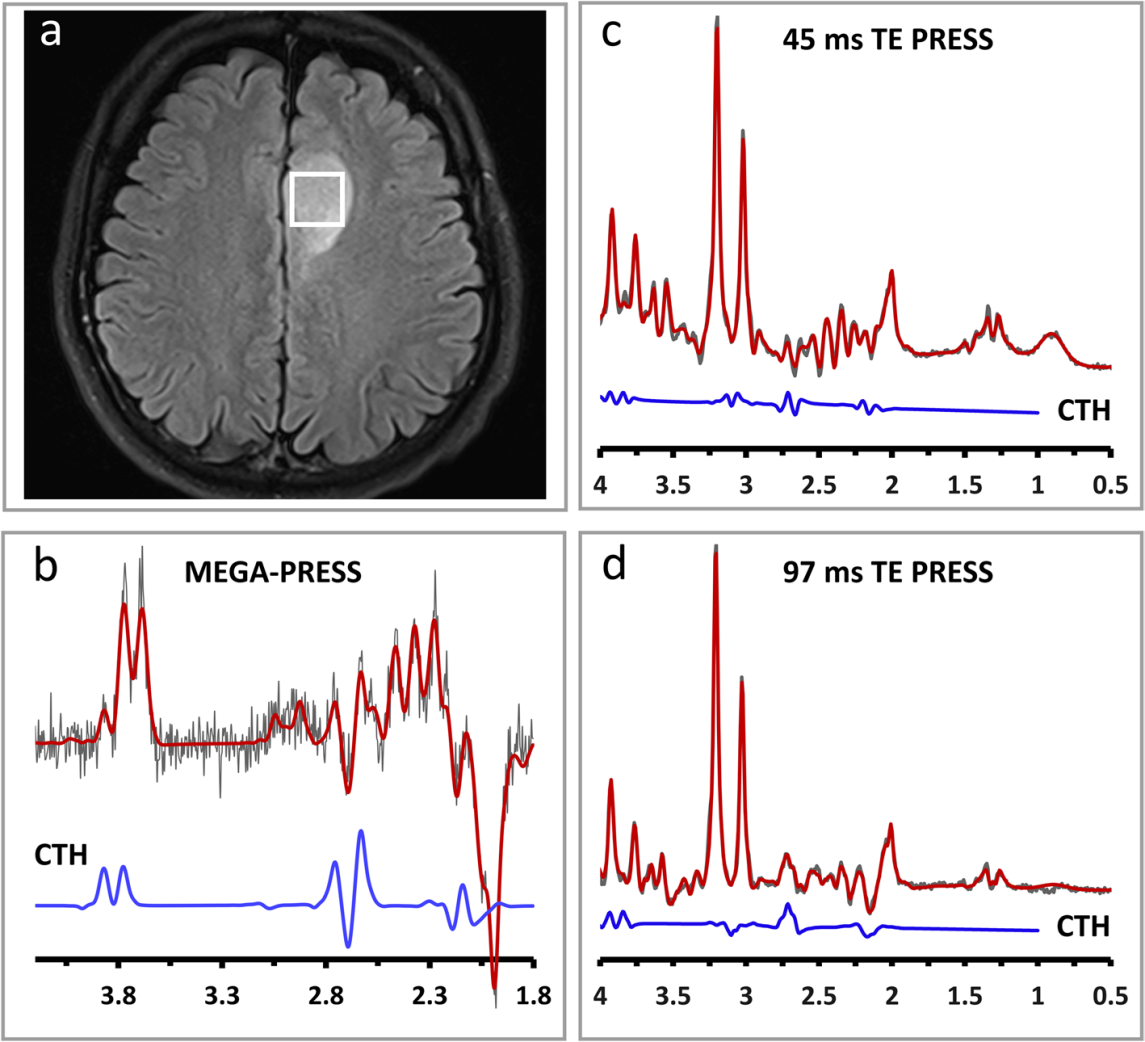
Representative spectra from a 1p/19q codeleted glioma patient. Panel **a** shows the location of the voxel. Cystathionine can be detected in MEGA-PRESS (concentration = 2.93 mM, CRLB = 7%), (**b**), 45 ms TE PRESS (concentration = 4.59 mM, CRLB = 14%), (**c**) and 97 ms TE PRESS (concentration = 6.46 mM, CRLB = 9%), (**d**). In panels **b**, **c** and **d**, the black spectrum shows the raw data, red spectrum is the fitted data, and the blue spectrum shows the cystathionine fit. CTH: cystathionine

The patient characteristics are summarized in Table 1. Representative spectra are shown in Fig. 3.

**Table 1:** Patient clinical and pathological characteristics

Characteristics	Number of Patients
Total number of patients	84
Median age (interquartile range) at the time of scan, y	53 (42–57)
Sex	
Male	45
Female	39
Histological diagnosis	
Glioma	67
Grades	
I	1
II	24
III	14
IV	28
IDH mutated gliomas	
Immunohistochemistry (IDH1 R132H)	22
Gene sequencing	14
Total	23
1p/19q codeletion status	
1p/19q codeletion	9
1p/19q non-codeletion	44
Lymphoma	2
Metastasis	4
Gliosis	4
Demyelination	4
Parasitic infection	1
Anaplastic meningioma	1
Arachnoid cyst	1

### Comparison of 45 ms and 97 ms TE PRESS for cystathionine detection in vivo

**Fig. 4:**
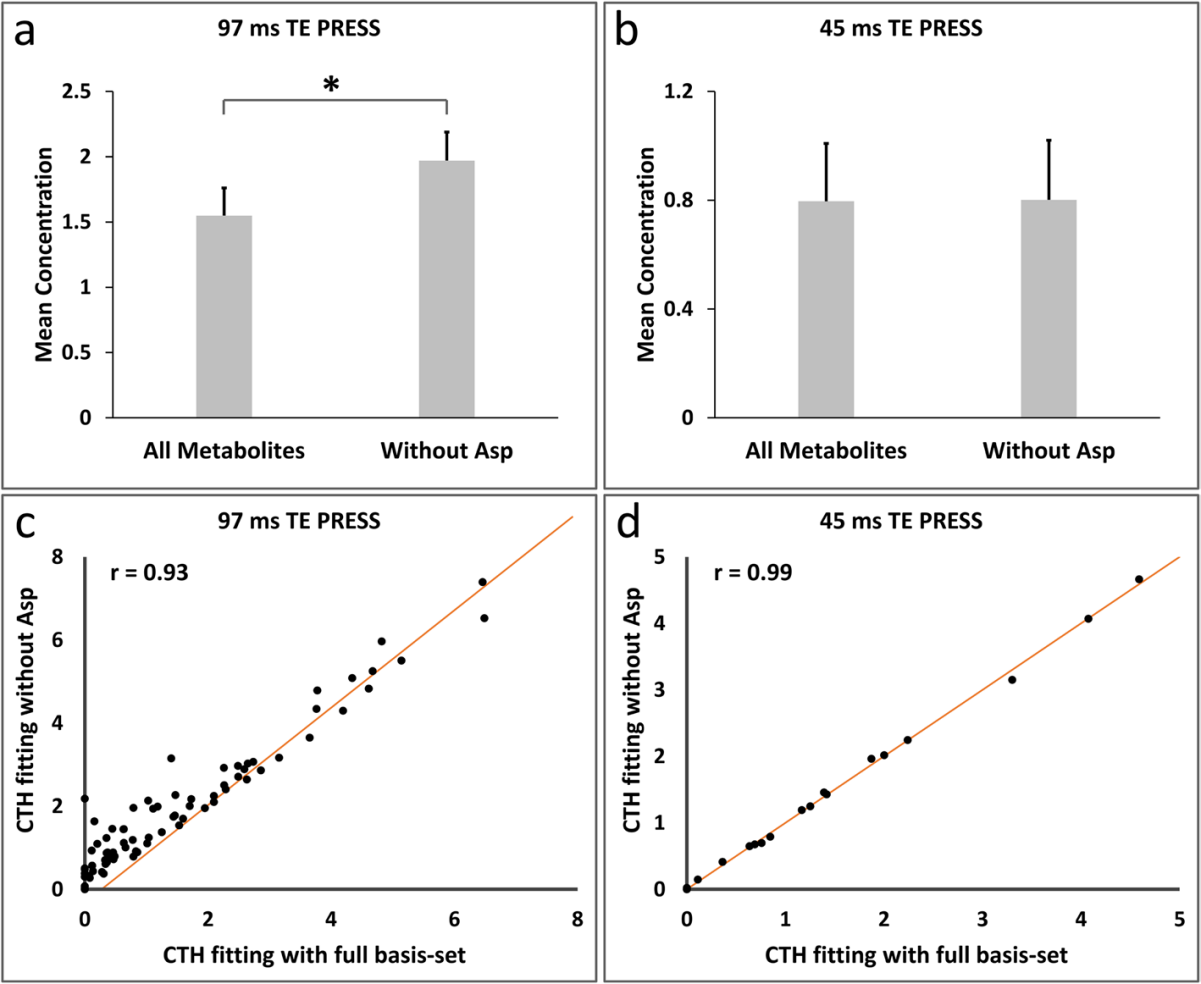
Comparison of 45 ms and 97 ms TE PRESS for cystathionine detection in vivo. The mean concentrations of cystathionine fitting with omission of aspartate are significantly higher than those fitting with full basis-set for 97 ms TE PRESS (*p* < 0.01), **a**), but not significantly different for 45 ms method (*p* = 0.49), **b**). The correlation coefficients between the cystathionine concentrations are 0.93 (*p* < 0.01), **c**) vs. 0.99 (*p* < 0.01), **d**) for 97 ms and 45 ms TE PRESS, respectively. Error bars in panel a and b replicate standard error. CTH: cystathionine; Asp: aspartate

For 97 ms TE PRESS, the mean concentrations of cystathionine fitting with omission of aspartate were significantly higher than those fitting with full basis-set (*n* = 84, 1.97 ± 2.01 mM vs. 1.55 ± 1.95 mM, paired t-test, *p* < 0.01, Fig. 4a), with the mean concentration difference of 0.42 ± 0.42 mM and ratio of the mean values of 1.27. For 45 ms TE PRESS, the mean concentrations of cystathionine obtained without and with aspartate in the basis-set were 0.801 ± 1.217 mM and 0.796 ± 1.217 mM, respectively (Fig. 4b). The concentrations were not significantly different (*n* = 34, paired t-test, *p* = 0.494). The mean difference of cystathionine concentrations was 0.005 ± 0.041 mM, and the ratio of the mean values was 1.006. Of note, the mean cystathionine concentration difference of 45 ms TE PRESS was significantly lower than that of 97 ms TE PRESS (*n* = 34 and 84, respectively; student t-test, *p* < 0.01). The correlation coefficients between the cystathionine concentrations were 0.93 (*p* < 0.01) vs. 0.99 (*p* < 0.01) for 97 ms TE and 45 ms TE PRESS, respectively (Fig. 4c and d).

A direct comparison was performed in 34 patients who had both 97ms and 45 ms TE PRESS spectra available. The results showed that the cystathionine concentrations derived without aspartate were higher than those with aspartate for both methods (1.90 ± 1.73 mM vs. 1.61 ± 1.62 mM, *p* < 0.01, and 0.801 ± 1.217 mM vs. 0.796 ± 1.217 mM, *p* = 0.494 for 97 ms and 45 ms TE PRESS, respectively), but only the outcomes of 97 ms TE PRESS reached statistical significance. The mean concentration difference of cystathionine was significantly higher in 97 ms TE PRESS than those in 45 ms TE PRESS (0.29 ± 0.27 mM vs. 0.005 ± 0.041 mM, paired t-test, *p* < 0.01).

**Fig. 5:**
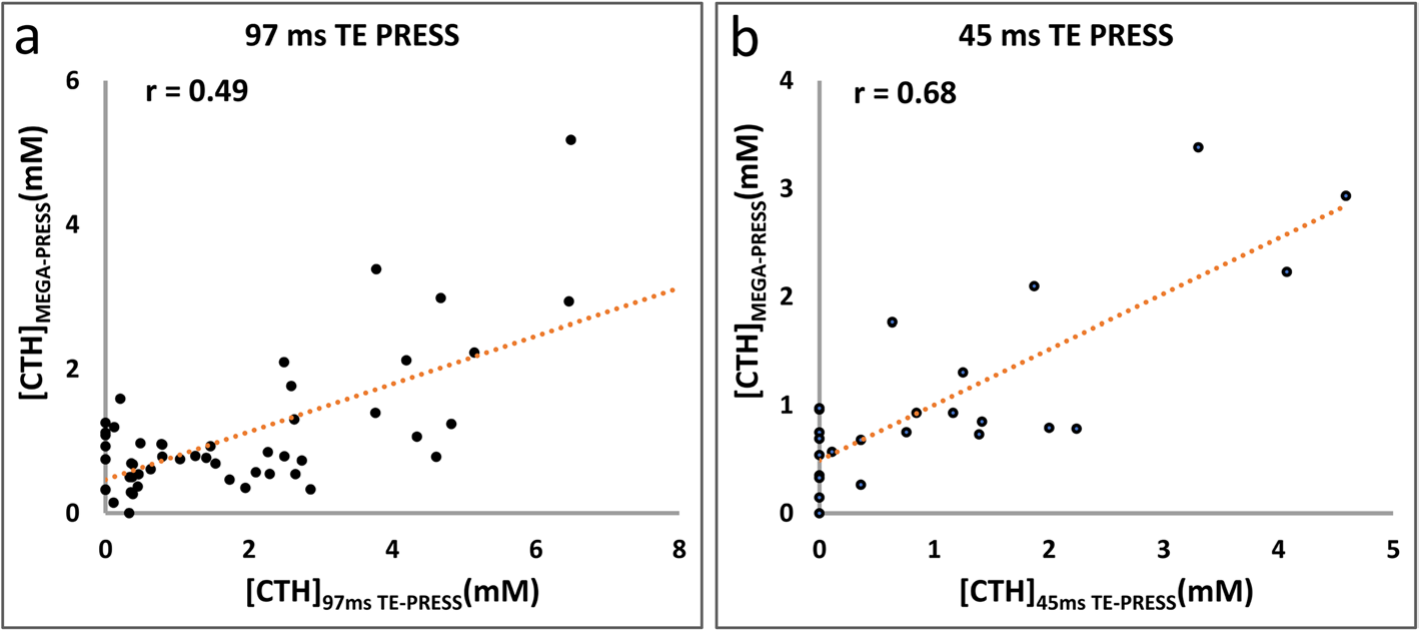
Correlations between cystathionine concentrations quantified with PRESS and MEGA-PRESS. The 45 ms TE PRESS data (*r* = 0.68, *p* < 0.01), **b**) is better correlated with those of MEGA-PRESS than 97 ms TE PRESS measurements (*r* = 0.49, *p* < 0.01), **a**). The cystathionine concentrations from PRESS are obtained with full basis sets. CTH: cystathionine

Both cystathionine concentrations of 97 ms and 45 ms TE PRESS obtained with full basis-sets showed significant correlation with those of MEGA-PRESS, with the correlation coefficients of 0.49 (*n* = 53) and 0.68 (*n* = 27), respectively (Fig. 5). Note that the 45 ms TE PRESS data was better correlated with those of MEGA than 97 ms TE PRESS measurements. Correlation analysis performed in a subgroup of patients with spectra of all 3 methods available (*n* = 27) showed the same results, with the correlation coefficients of 0.56 and 0.68 for 97 ms and 45 ms TE PRESS data respectively.

### Comparison of cystathionine concentrations in different patient cohorts

We compared the cystathionine concentrations as well as the corresponding CRLBs of 1p/19q-codeleted gliomas, 1p/19q non-codeleted gliomas and other tumors. No significant difference were observed in cystathionine levels or the CRLBs between all 3 groups for 45ms TE PRESS (*n* = 3, 15, and 4 for each subgroup), 97 ms TE PRESS (*n* = 9, 44, and 17) or MEGA PRESS (*n* = 7, 22, and 11). The detailed information are shown in Additional File 2.

Considering a cystathionine threshold of 1mM concentration or 25% CRLB reported previously [7], the sensitivity and specificity for identifying 1p/19q codeletion were 66.7% (2/3) and 73.7% (14/19) for 45 ms TE PRESS, and 44.4% (4/9) and 52.5% (32/61) for 97 ms TE PRESS, respectively (Table 2). Direct comparison was also performed in patients with both 45 ms and 97 ms TE PRESS available (*n* = 22), and the results showed that the sensitivity and specificity for 1p/19q-codeleted gliomas were 66.7% (2/3) and 73.7% (14/19) for 45 ms TE PRESS, and 66.7% (2/3) and 47.4% (9/19) for 97 ms TE PRESS, respectively (as shown in Additional File 3).

**Table 2:** The sensitivity and specificity of PRESS for 1p/19q codeletion

Sequences	Number of Patients^a^	Sensitivity	Specificity	Accuracy
45 ms TE PRESS	22	66.7% (2/3)	73.7% (14/19)	72.7% (16/22)
97 ms TE PRESS	70	44.4% (4/9)	52.5% (32/61)	51.4% (36/70)

^a^Only glioma patients with gene sequencing results available and other tumors were included in analysis

## Discussion

In this paper, we reported a comparative study of short and long TE PRESS sequences, focusing on cystathionine detection in gliomas. Our results demonstrated that PRESS sequence with the optimized total echo time of 45 ms yielded more precise and robust cystathionine estimates than the widely-used 97 ms TE method.

The 45 ms TE PRESS sequence outperforms the 97 ms TE approach regarding cystathionine detectability in several aspects. Considering the T_2_ relaxation effects, short TE permits the cystathionine signal to be acquired with minimal T_2_ signal loss, and thus would be more preferable for accurate quantification of cystathionine concentrations. More importantly, the 45 ms echo time generates a well-defined cystathionine peak against a relatively flat aspartate peak around 2.72 ppm, where the maximum multiples of cystathionine are present, leading to improved differentiation between the 2 compounds. These were further validated by in vivo study showing a significantly lower difference of cystathionine concentrations obtained without and with aspartate in the basis set in 45 ms TE PRESS, suggesting more precise assignment of cystathionine and aspartate signals during fitting process. In contrast, the 97 ms TE PRESS results exhibited a 1.272-fold rise in cystathionine levels when aspartate was excluded from the basis set, implying a misrecognition of these 2 metabolites by LCModel. This aligns with prior findingsby Branzoli et al. where aspartate showed a 2.69-fold higher increase in concentrations by omission of cystathionine from the spectral analysis in 97 ms TE PRESS [5]. 

Cystathionine concentrations of 45 ms TE PRESS had a better correlation with those quantified using MEGA-PRESS, as compared to the 97 ms TE counterpart. MEGA-PRESS sequence allowed the generation of an edited cystathionine H4 multiplet at 2.72 ppm, while canceling aspartate resonances which were not affected by the editing pulses, and thus served as a standard with respect to cystathionine concentrations in our study [3]. The higher correlation further verified the advantage of 45 ms TE PRESS over the 97 ms TE method regarding cystathionine measurements.

Of note, significantly higher cystathionine concentrations were not observed in spectra of both PRESS and MEGA-PRESS for 1p/19q-codeleted gliomas, compared to 1p/19q non-codeleted gliomas or other tumors. Branzoli et al. reported significantly higher cystathionine levels in IDH-mutant 1p/19q-codeleted gliomas versus IDH-mutant 1p/19q non-codeleted gliomas using MEGA-PRESS and 97 ms TE PRESS [3, 7]. The discrepancy between the 2 studies might be partly attributed to the small size of 1p/19q-codeleted glioma patients in our cohorts. Nine of the 53 glioma patients who had gene sequencing showed positive 1p/19q codeletion, with less codeleted patients having available spectra for 45ms TE PRESS (*n* = 3) or MEGA-PRESS (*n* = 7). The limited sample size might have hindered the detection of a distinct pattern in cystathionine levels across various patient cohorts. Additionally, not including IDH status as a covariate in the analysis might also contribute to the inconsistency in the results. Our study compared 1p/19q-codeleted gliomas to 1p/19q non-codeleted gliomas including both IDH-mutated 1p/19q non-codeleted and IDH wild-type gliomas. In contrast, Branzoli et al’s study primarily categorized the patient population into IDH-mutated 1p/19q-codeleted and IDH-mutated 1p/19q non-codeleted groups. Note that high cystathionine concentration was also observed in 1 of the 2 IDH wild-type glioma patients in their study [3]. We didn’t perform subgroup analysis due to small number of IDH-mutated glioma patients (*n* = 23).

We therefore were not able to observe a good diagnostic performance of cystathionine for 1p/19q codeletion with 45ms TE or 97 ms TE PRESS. Compared to Branzoli et al’s study [7], both the 45 ms TE and 97 ms TE PRESS yielded a comparable specificity (73.7% and 52.5% vs. 61%), but a lower sensitivity (66.7% and 44.4% vs. 92%). This discrepancy raised several questions in this field: (1) what the diagnostic strategy should be, identifying IDH mutation first and then 1p/19q codeletion or 1p/19q codeletion directly; (2) how the diagnostic performance of PRESS was compared to MEGA-PRESS; (3) how the MRS sequences should be tailored in clinical settings for genotyping purpose in gliomas; and (4) what the cutoff values of cystathionine should be for PRESS and MEGA-PRESS. Those questions warranted further investigations into the diagnostic efficacy of cystathionine MRS for 1p/19q-codeleted gliomas, particularly in the context of larger cohort studies.

Although the diagnostic role of MRS for 1p/19q codeletion remained uncertain, our findings suggested the potential of optimized 45 ms TE PRESS sequence for improving the diagnostic utility of MRS for this genetic alteration with a better diagnostic accuracy. Coupled with detection of 2HG for IDH mutations, MRS could thus more accurately categorized adult-type diffuse gliomas [17]. On the other hand, our approach would enhance the monitoring of treatment response by offering an accurate and noninvasive way of repeatedly assessing cystathionine levels. This would subsequently facilitate better clinical management in 1p/19q-codeleted glioma patients. In summary, our data provided evidence for MRS scheme tailoring, specifically integrating the 45 ms TE PRESS sequence into clinical practice.

Our study is limited by a small sample size of 1p/19q-codeleted glioma patients as mentioned above, although we have a relatively large patient population in the in vivo trial. However, the low rate of 1p/19q-codeleted patients is partly due to the prospective nature of our in vivo study. Secondly, not all patients performed 45 ms TE PRESS and MEGA-PRESS. Thirdly, our study is also limited by lack of corroboration of the in vivo results by ex vivo tissue analysis methods such as liquid chromatography-mass spectroscopy in tumor samples. Further studies employing such methods will not only facilitate direct comparison of different spectroscopic sequences in terms of cystathionine detection, but also provide a better understanding of cystathionine levels in different subtype of gliomas.

In conclusion, the optimized TE = 45 ms PRESS sequence better resolves cystathionine signals from the adjacent aspartate resonances, allowing cystathionine to be more precisely measured. Cystathionine detectability in vivo is anticipated to become a promising tool for noninvasive classification of gliomas following 2-hydroxyglutarate, and the ability to provide accurate estimates will be of great importance to increase the clinical potential of this special metabolite.

## Supplementary Information

Additional file 1. Comparison of the cystathionine concentrations (a) as well as the corresponding CRLBs (b) of 1p/19q codeleted gliomas, 1p/19q non-codeleted gliomas and other tumors.

Additional file 2. Multi-sequence MRSinMRS checklist.

Additional file 3. The sensitivity and specificity of PRESS for 1p/19q codeletion in patients with both 45 ms and 97 ms TE spectra available.

Additional file 4. 

## Data Availability

The datasets used during the current study are available from the corresponding author on reasonable request.

## Abbreviations

TE: Echo time
PRESS: Point-resolved spectroscopy
MEGA-PRESS: Mescher-Garwood point-resolved spectroscopy
FWHM: Full width at half maximum
CRLB: Cramer-Rao lower bounds
