# Correction: Optimization and validation of echo times of point-resolved spectroscopy for cystathionine detection in gliomas

**DOI:** 10.1186/s40644-024-00806-4

**Published:** 2024-11-21

**Authors:** Min Zhou, Zhuang Nie, Jie Zhao, Yao Xiao, Xiaohua Hong, Yuhui Wang, Chengjun Dong, Alexander P. Lin, Ziqiao Lei

**Affiliations:** 1 Department of Radiology, Union Hospital, Tongji Medical College, Huazhong University of Science and Technology, Wuhan, Hubei China; 2 Center for Clinical Spectroscopy, Brigham and Women’s Hospital, Harvard Medical School, Boston, MA USA; 3 Tumor Center, Union Hospital, Tongji Medical College, Huazhong University of Science and Technology, Wuhan, Hubei China

**Correction** **:** **Cancer Imaging 24, 118 (2024)**

**https://doi.org/10.1186/s40644-024-00764-x**

Following publication of the original article [1], the authors identified an error in Figure 2C, where "Gly fits" should be corrected to "Asp fits".

Originally published figure 2:


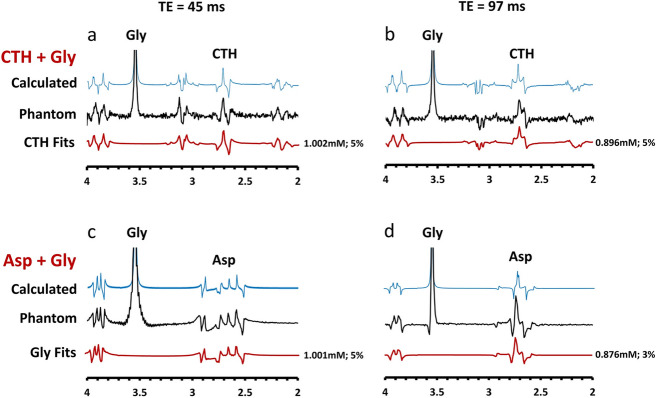


Corrected figure 2:


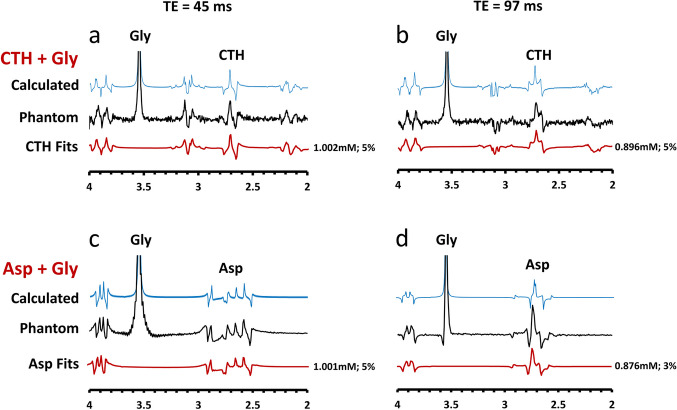


The original article has been corrected.
