# Supplementary material for: Optimization and validation of echo times of point-resolved spectroscopy for cystathionine detection in gliomas
Source: Cancer Imaging. 2024 Sep 2;24:118. doi: 10.1186/s40644-024-00764-x (PMC11367870; doi:10.1186/s40644-024-00764-x)
Supplement: Supplementary file 2 — Additional file 2. Multi-sequence MRSinMRS checklist. [file 40644_2024_764_MOESM2_ESM.docx]

Additional Table 1. Multi-sequence MRSinMRS checklist

| 1. Hardware |  |  |  |
| --- | --- | --- | --- |
| a. Field strength (tesla) | 3T | 3T | 3T |
| b. Manufacturer | Siemens | Siemens | Siemens |
| c. Model (software version if available) | Skyra (VE11C) | Skyra (VE11C) | Skyra (VE11C) |
| d. RF coils: nuclei, number of channels, type, body part | 32 channel head coil | 32 channel head coil | 32 channel head coil |
| e. Additional hardware | N/A | N/A | N/A |
| 2. Acquisition |  |  |  |
| a. Pulse sequence (name, bandwidth in Hz, number of spectral points) | PRESS (2000 Hz, 1024 data points) | PRESS (2000 Hz, 1024 data points) | MEGA-PRESS (2000 Hz, 2048 data points) |
| b. Volume of Interest (VOI) locations | Including as much of the lesion as possible while avoiding cystic, hemorrhagic, or necrotic regions | The same VOI as the previous sequence | The same VOI as the previous sequence |
| c. Nominal VOI size [cm3, mm3] | Varying across cases | The same VOI size as the previous sequence | The same VOI size as the previous sequence |
| d. Repetition Time (TR), Echo Time (TE) [ms, s] | TR/TE = 2000/45 ms, | TR/TE = 2000/97 ms | TR/TE = 2000/68 ms |
| e. Total number of Excitations or acquisitions per spectrum  In time series for kinetic studies   1. Number of Averaged spectra (NA) per time-point 2. Averaging method (e.g. block-wise or moving average) 3. Total number of spectra (acquired / in time-series) | 128 averages | 128 averages | 128 pairs of scans |
| f. Additional sequence parameters  If STEAM:, Mixing Time (TM)  If MRSI: 2D or 3D, FOV, Matrix matrix size, acceleration factor, sampling method | None | None | Editing pulse (duration: 19.2 ms; bandwidth: 62 Hz; offset frequency: -2.0ppm) was applied at 1.9 ppm for the edit-on condition and at 7.5 ppm for the edit-off condition, in an interleaved fashion |
| g. Water Suppression Method | Outer volume suppression | Outer volume suppression | Variable power with optimized relaxation delays and outer volume suppression |
| h. Shimming Method, reference peak, and thresholds for “acceptance of shim” chosen | Vendor-provided automated shimming followed by manual shimming of water to < 14 Hz | Vendor-provided automated shimming followed by manual shimming of water to < 14 Hz | Vendor-provided automated shimming followed by manual shimming of water to < 14 Hz |
| i. Triggering or motion correction method  (respiratory, peripheral, cardiac triggering, incl. device used and delays) | None | None | None |
| 3. Data analysis methods and outputs |  |  |  |
| a. Analysis software | LCModel vers 6.3-1R | LCModel vers 6.3-1R | LCModel vers 6.3-1R |
| b. Processing steps deviating from quoted reference or product | OpenMRSLab preprocessing prior to use of LCModel | OpenMRSLab preprocessing prior to use of LCModel | FID-A preprocessing prior to use of LCModel |
| c. Output measure  (e.g. absolute concentration, institutional units, ratio)Processing steps deviating from quoted reference or product | Absolute concentration and CRLB | Absolute concentration and CRLB | Absolute concentration and CRLB |
| d. Quantification references and assumptions, fitting model assumptions | Water reference with the assumed water concentration of 55.5 mM; Simulated basis-set containing 25 metabolites, which included cystathionine, alanine, ascorbate, aspartate, beta-hydroxybutyrate, 2-hydroxyglutyrate, creatine, phosphocreatine, ethanolamine, γ-aminobutyric acid, glucose, glutamine, glutamate, glycine, glutathione, myo-inositol, N-acetylaspartate, N-acetyl-aspartyl-glutamate, phosphocholine, glycerophosphocholine, lactate, serine, phosphoethanolamine, scyllo-inositol and taurine | Water reference with the assumed water concentration of 55.5 mM; Simulated basis-set containing 25 metabolites as the previous sequence | Water reference with the assumed water concentration of 55.5 mM; Simulated basis-set containing 8 metabolites, consisting of 2-hydroxyglutyrate, cystathionine, γ-aminobutyric acid, glutamate, glutamine, glutathione, N-acetylaspartate and N-acetyl-aspartyl-glutamate |
| 5. Data Quality |  |  |  |
| a. Reported variables  (SNR, Linewidth (with reference peaks)) | SNR: 29.1 ± 14.2  FWHM: 0.034 ± 0.013 ppm | SNR: 25.7 ± 9.7  FWHM: 0.038 ± 0.015 ppm | SNR: 6.5 ± 3.4  FWHM: 0.049 ± 0.020 ppm |
| b. Data exclusion criteria | PRESS spectra with an SNR < 5 or FWHM of Cr peak > 0.143 ppm were excluded due to poor quality | PRESS spectra with an SNR < 5 or FWHM of Cr peak > 0.143 ppm were excluded due to poor quality | Spectra were visually inspected and excluded if noise obscured the spectra (other than NAA) such that it could not be reliably fitted |
| c. Quality measures of postprocessing Model fitting (e.g. CRLB, goodness of fit, SD of residual) | CRLB of NAA: 8.7 ± 11.9% | CRLB of NAA: 8.7 ± 8.2% | CRLB of NAA: 7.4 ± 3.3% |
| d. Sample Spectrum | Figure 3c | Figure 3d | Figure 3b |
