# Supplementary material for: Optimization and validation of echo times of point-resolved spectroscopy for cystathionine detection in gliomas
Source: Cancer Imaging. 2024 Sep 2;24:118. doi: 10.1186/s40644-024-00764-x (PMC11367870; doi:10.1186/s40644-024-00764-x)
Supplement: Supplementary file 3 — Additional file 3. The sensitivity and specificity of PRESS for 1p/19q codeletion in patients with both 45 ms and 97 ms TE spectra available. [file 40644_2024_764_MOESM3_ESM.docx]

Additional Table 2. The sensitivity and specificity of PRESS for 1p/19q codeletion in patients with both 45 ms and 97 ms TE spectra available

| **Sequences** | **Number of Patients^[[1]](#footnote-1)^** | **Sensitivity** | **Specificity** | **Accuracy** |
| --- | --- | --- | --- | --- |
| 45 ms TE PRESS | 22 | 66.7% (2/3) | 73.7% (14/19) | 72.7% (16/22) |
| 97 ms TE PRESS | 22 | 66.7% (2/3) | 47.4% (9/19) | 50.0% (11/22) |

1. Only glioma patients with gene sequencing results available and other tumors were included in analysis. [↑](#footnote-ref-1)
