# Supplementary material for: Optimization and validation of echo times of point-resolved spectroscopy for cystathionine detection in gliomas
Source: Cancer Imaging. 2024 Sep 2;24:118. doi: 10.1186/s40644-024-00764-x (PMC11367870; doi:10.1186/s40644-024-00764-x)
Supplement: Supplementary file 4 — Additional file 4. [file 40644_2024_764_MOESM4_ESM.docx]

table S1 The Shapiro-Wilk Test between over 14 years old and under 14 years old in the flatfoot

| position | Variable | Age | Shapiro–Wilk test | | |
| --- | --- | --- | --- | --- | --- |
|  |  |  | Statistic | df | Sig. |
| Hindfoot | calcaneofibular distance | ＜14 | 0.898 | 20 | 0.039 |
|  |  | ≥14 | 0.980 | 42 | 0.647* |
|  | talofibular distance | ＜14 | 0.871 | 20 | 0.012 |
|  |  | ≥14 | 0.982 | 42 | 0.749* |
|  | CIA_S | ＜14 | 0.925 | 20 | 0.122* |
|  |  | ≥14 | 0.980 | 42 | 0.645* |
|  | CIA_T | ＜14 | 0.953 | 20 | 0.419* |
|  |  | ≥14 | 0.948 | 42 | 0.053* |
|  | LTCA_C | ＜14 | 0.750 | 20 | 0.000 |
|  |  | ≥14 | 0.916 | 42 | 0.004 |
|  | LTCA_S | ＜14 | 0.843 | 20 | 0.004 |
|  |  | ≥14 | 0.955 | 42 | 0.100* |
|  | LTCA_T | ＜14 | 0.899 | 20 | 0.039 |
|  |  | ≥14 | 0.929 | 42 | 0.012 |
|  | TACA_C | ＜14 | 0.900 | 20 | 0.040 |
|  |  | ≥14 | 0.919 | 42 | 0.006 |
|  | TACA_S | ＜14 | 0.951 | 20 | 0.389* |
|  |  | ≥14 | 0.954 | 42 | 0.091* |
|  | TACA_T | ＜14 | 0.974 | 20 | 0.841* |
|  |  | ≥14 | 0.983 | 42 | 0.781* |
|  | TCA_C | ＜14 | 0.903 | 20 | 0.046 |
|  |  | ≥14 | 0.906 | 42 | 0.002 |
|  | TCA_S | ＜14 | 0.950 | 20 | 0.362* |
|  |  | ≥14 | 0.960 | 42 | 0.145* |
|  | TCA_T | ＜14 | 0.981 | 20 | 0.943* |
|  |  | ≥14 | 0.989 | 42 | 0.961* |
|  | Sinus tarsi volume | ＜14 | 0.967 | 20 | 0.695* |
|  |  | ≥14 | 0.962 | 42 | 0.179* |
| Midfoot | MCH | ＜14 | 0.955 | 20 | 0.455* |
|  |  | ≥14 | 0.974 | 42 | 0.453* |
|  | TNCA_C | ＜14 | 0.915 | 20 | 0.081* |
|  |  | ≥14 | 0.949 | 42 | 0.058* |
|  | TNCA_S | ＜14 | 0.931 | 20 | 0.160* |
|  |  | ≥14 | 0.955 | 42 | 0.097* |
|  | TNCA_T | ＜14 | 0.914 | 20 | 0.077* |
|  |  | ≥14 | 0.979 | 42 | 0.618* |
| Forefoot | Hibb angle_C | ＜14 | 0.956 | 20 | 0.468* |
|  |  | ≥14 | 0.866 | 42 | 0.000 |
|  | Hibb angle_S | ＜14 | 0.969 | 20 | 0.735* |
|  |  | ≥14 | 0.916 | 42 | 0.005 |
|  | Hibb angle_T | ＜14 | 0.945 | 20 | 0.297* |
|  |  | ≥14 | 0.946 | 42 | 0.048 |
|  | Meary’s angle_C | 0.912 | 20 | 0.068 | 0.912* |
|  |  | 0.974 | 42 | 0.458 | 0.974* |
|  | Meary’s angle_S | 0.972 | 20 | 0.805 | 0.972* |
|  |  | 0.761 | 42 | 0.000 | 0.761* |
|  | Meary’s angle T | 0.867 | 20 | 0.010 | 0.867* |
|  |  | 0.886 | 42 | 0.001 | 0.886* |

* indicates that the data conform to a normal distribution
